# Efficacy of anti-VEGF agents in the treatment of elderly hepatocellular carcinoma: a systematic review

**DOI:** 10.18632/oncotarget.21452

**Published:** 2017-10-03

**Authors:** Xiaofei Li, Daofu Zhang, Shan Guan, Weiwei Ye, Liwen Liu, Lianqing Lou

**Affiliations:** ^1^ Department of Infectious Diseases, Yi Wu Central Hospital, Zhejiang Province, 322000, China; ^2^ Liao Cheng City People's Hospital, Shandong Province, 252000, China; ^3^ Department of Infectious Diseases, Liaocheng People's Hospital, Shandong Province, 252000, China

**Keywords:** hepatocellular carcinoma, elderly, randomized controlled trials, meta-analysis

## Abstract

**Purpose:**

We aimed to investigate the role of anti-vascular endothelial growth factor (VEGF) agents, including tyrosine-kinase inhibitors or monoclonal anti-bodies, in the treatment of elderly hepatocellular carcinoma (HCC) patients.

**Materials and Methods:**

Databases from PubMed, Web of Science and abstracts presented at ASCO meeting up to March 31, 2017 were searched to identify relevant studies. The endpoints were overall survival (OS) and progression-free survival (PFS). Data were examined using age cutoffs of 65 years.

**Results:**

A total of 1,309 elderly (aged ≥ 65 years) HCC patients from seven trials were included for analysis. Our results demonstrated that the use of anti-VEGF agents MTAs in patients aged ≥ 65 years significantly improved PFS (HR 0.65, 95% CI: 0.55–0.76, *p* < 0.001) but not for OS (HR 0.87, 95% CI: 0.73–1.05, *p* = 0.15). Sub-group analysis according to treatment line showed that the use of anti-VEGF agents as second-line treatment significantly improved PFS (HR 0.55, 95% CI: 0.45–0.67, *p* < 0.001) and marginally improved OS (HR 0.83, 95% CI: 0.68–1.01, *p* = 0.061). Additionally, no survival benefits were observed in elderly HCC received first-line anti-VEGF treatments in terms of PFS (HR 0.87, 95% CI: 0.67–1.13, *p* = 0.29) and OS (HR 1.19, 95% CI: 0.74–1.36, *p* = 0.47). No publication bias was detected by Begg's and Egger's tests for OS.

**Conclusions:**

The findings of this study show that elderly HCC patients who relapsed after a first-line sorafenib treatment obtains a survival benefits from anti-VEGF agents rechallenge. Further studies are recommended to search for predictors of good responders in these patients received anti-VEGF agents.

## INTRODUCTION

Hepatocellular carcinoma (HCC) is one of the leading causes of cancer-related deaths with an estimated 748,300 new liver cancer cases and 695,900 cancer deaths occurred worldwide [1, 2]. Surgical resection and liver transplantation are considered the only potentially curative treatment for HCC patients. However, more than 70% of HCC patients present with intermediate-stage or advanced-stage disease at the time of diagnosis, and therefore are not suitable for surgical resection [3]. The prognosis of advanced HCC patients is dismal with a median overall survival time of about 7 months [4]. Novel treatments for HCC patients are clearly needed. Additionally, HCC usually develops in patients with hepatitis B virus infection, hepatitis C virus infection, or alcoholic liver disease, which develops over a long period of time [5, 6]. Additionally, the widely use of anti-viral therapy might further delay the development of HCC. As a result, HCC is commonly diagnosed in middle-aged and elderly populations, and management of elderly HCC patients is becoming a global issue [5, 7].

Angiogenesis, the formation of new blood vessels, is known to play a central role in the progression of many solid tumors, including HCC [8–10]. Among the many mediators of new blood vessel formation, vascular endothelial growth factor (VEGF) family of ligands plays a primary role [11, 12]. Inhibition of VEGF signaling pathway has proven an effective strategy for the treatment of HCC patients [13] and other solid tumors [14–18]. Until now, sorafenib is the only systematic treatment approved by FDA for use in advanced HCC patients [19–21]. Additionally, several novel anti-VEGF agents have been extensively assessed in many prospective clinical trials [22–25]. However, as the stringent enrolment criteria for patients in prospective trials, the enrolled elderly patients in clinical studies are not entirely representative of the overall elderly patient population. In addition, treatment of elderly HCC patients may be complicated by several comorbid conditions and greater concomitant medication use when compared to younger patients [26, 27]. As a result, clinical data obtained from a selected elderly population cannot be automatically extrapolated to the great majority of non-selected elderly HCC patients.

Currently, there is still no general agreement on the definition of the elderly population. Most developed countries accept the chronological age of 65 years as the definition of an elderly person. In the present, we agree with the cut-off of ≥ 65 years to refer to the older population. As the elderly HCC population increases, it is urgently needed to define the best treatment strategy for these patients. We thus perform the present study to assess the efficacy of anti-VEGF agents in the treatment of elderly HCC patients.

## MATERIALS AND METHODS

### Search strategy

A comprehensive search for relevant articles was conducted in databases including the Pubmed, Embase and the Cochrane Library electronic databases (Supplementary Table 1). The date of the last search was 31 January 2017. Articles with the following test words in their titles, abstracts or keywords were examined: “anti-VEGF agents”, “angiogenesis inhibitors”, “sorafenib”, “sunitinib”, “regorafenib”, “ramucirumab”, “axitinib”, “brivanib”, “hepatocellular carcinoma”, “randomized controlled trials”. An independent search of the Google scholar was also performed to ensure that no additional clinical trials had been overlooked. If more than one publication was found for the same trial, the most complete, recent, and updated report of the clinical trial was included. Ethical approval for this study was not unnecessary since it was a meta-analysis that collected and analyzed data from the existing literatures.

### Study selection

Clinical trials that met the following criteria were included: (1) randomized controlled phase II and III trials in patients with HCC; (2) participants assigned to treatment with or without anti-VEGF agents; (3) survival data of elderly patients were available;

### Data extraction

Two authors independently performed data extraction. This meta-analysis was conducted in accordance with the Preferred Reporting Items for Systematic review and Meta-Analysis (PRISMA) statement (Supplementary Table 2) [28]. Disagreements between investigators were resolved by discussion and consensus. A standardized Excel file was used for data extraction. The following data were extracted: first author, publication year, the number of enrolled patients and elderly patients, median age, hazard ratios (HRs) with 95% confidence intervals (CIs) for OS and PFS in elderly HCC patients.

### Clinical end point and statistical method

The outcome measures of interest were progression-free survival (PFS) and overall survival (OS). PFS and OS were considered as time-to-event variables, and therefore were expressed as HRs with 95% CIs for each study. HR > 1 reflected more deaths or progression in anti-VEGF-containing regimens, and vice versa. Heterogeneity across the studies was assess by using the χ^2^-based Q statistic [29]. The *I*^2^ statistic was also calculated to quantitatively evaluate the degree of inconsistency between trials. Predefined sub-group analysis according to treatment line was performed. We used the Begg and Egger tests to assess the presence of publication bias [30]. Study quality was roughly assessed by using the Jadad five-item scale [31]. All *p*-value of less than 0.05 was considered statistically significant. All statistical analysis was calculated using Version 2 of the Comprehensive MetaAnalysis program (Biostat, Englewood, NJ).

## RESULTS

**Figure 1:**
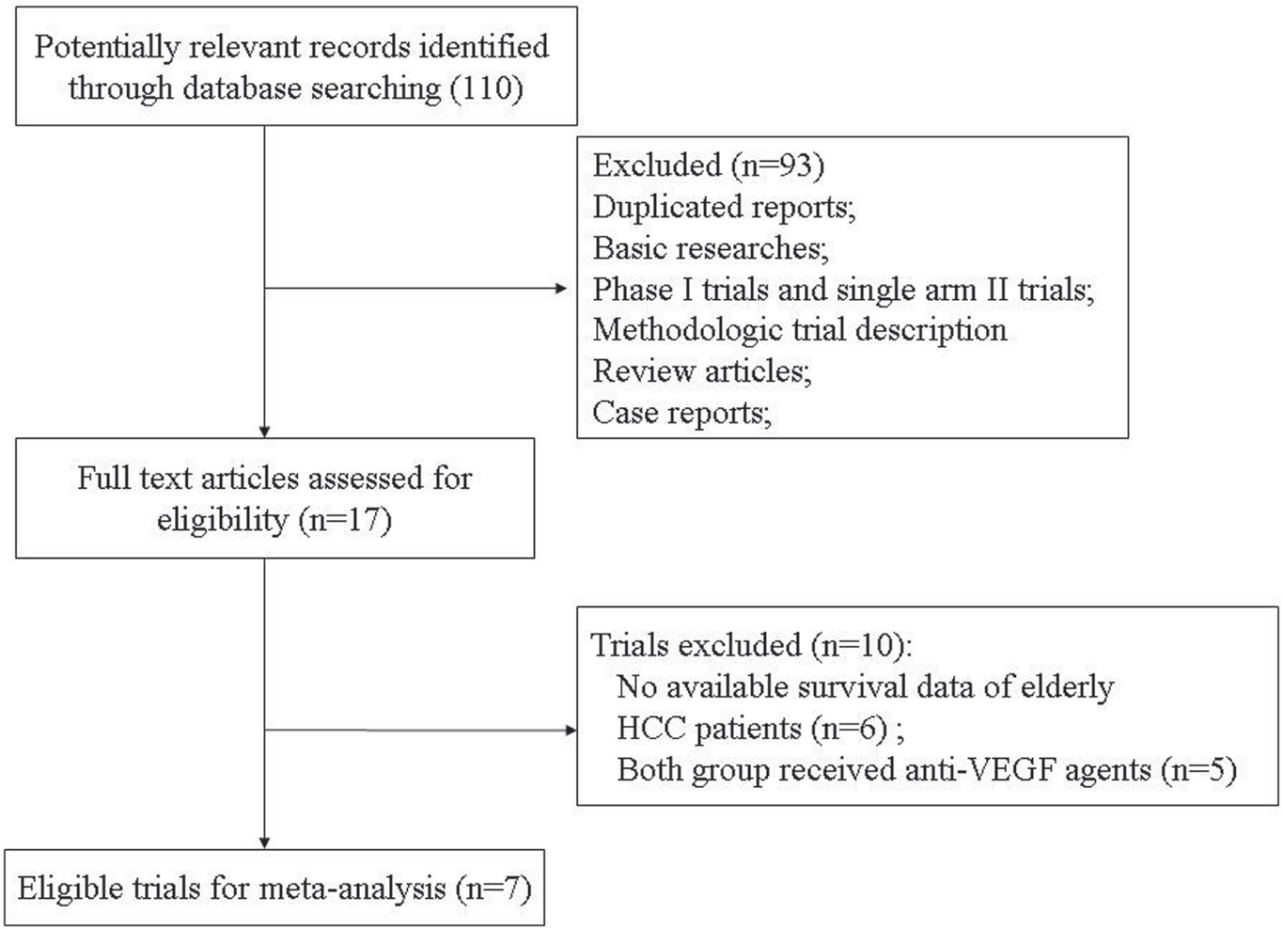
Studies eligible for inclusion in the meta-analysis

Our search strategy yielded 110 clinical studies related to anti-VEGF agents in HCC patients from databases. The reasons for study exclusion were shown in Figure 1. Finally, a total of seven prospective randomized controlled trials were considered eligible, including one phase II trials [32] and six phase III trials [33–38]. A total of 1,309 elderly (aged ≥ 65 years) HCC patients were included. The characteristics of patients and studies were listed in Table 1. The quality of each included study was roughly assessed according to Jadad scale, and the median Jadad score of the included studies was 5 (range 3–5).

**Table 1:** Baseline characteristics of seven included randomized controlled trials

Authors/year	Phase	Total	Cutoff of age	No. of patients	Treatment arms	median age (years)	median PFS, m	median OS, m	Jadad Score
Cheng AL, et al. 2009	III	271	≥ 65	32	Sorafenib 400 mg bid po	51	2.8	6.5	5
					placebo	52	1.4	4.2	
Kudo M, et al. 2011	III	458	≥ 65	152	Sorafenib 400 mg bid po + TACE	69	5.4	29.7	5
					Placebo + TACE	70	3.7	NR	
Kudo M, et al. 2014	III	502	≥ 65	159	Brivanib 800 mg qd po	57	12	26.4	5
					Placebo	59	10.9	26.1	
Bruix J, et al. 2015	III	1114	≥ 65	370	Sorafenib 400 mg bid po	58	8.5	NR	5
					Placebo	60	8.4	NR	
Kang YK, et al. 2015	II	202	≥ 65	85	Axitinib 5 mg bid po	61	3.6	12.7	3
					Placebo	63	1.9	9.7	
Zhu AX, et al. 2015. (REACH)	III	565	≥ 65	253	Ramucirumab 8 mg/kg	64	2.8	9.2	5
					Placebo	62	2.1	7.6	
Bruix J, et al. 2017	III	573	≥ 65	258	Regorafenib 160 mg po	64	3.1	10.6	5
					Placebo	62	1.5	7.8	

Abbreviations: OS, overall survival; PFS, progression-free survival; TACE, transcatheter arterial chemoembolization; NR, not reported.

### Progression-free survival

**Figure 2:**
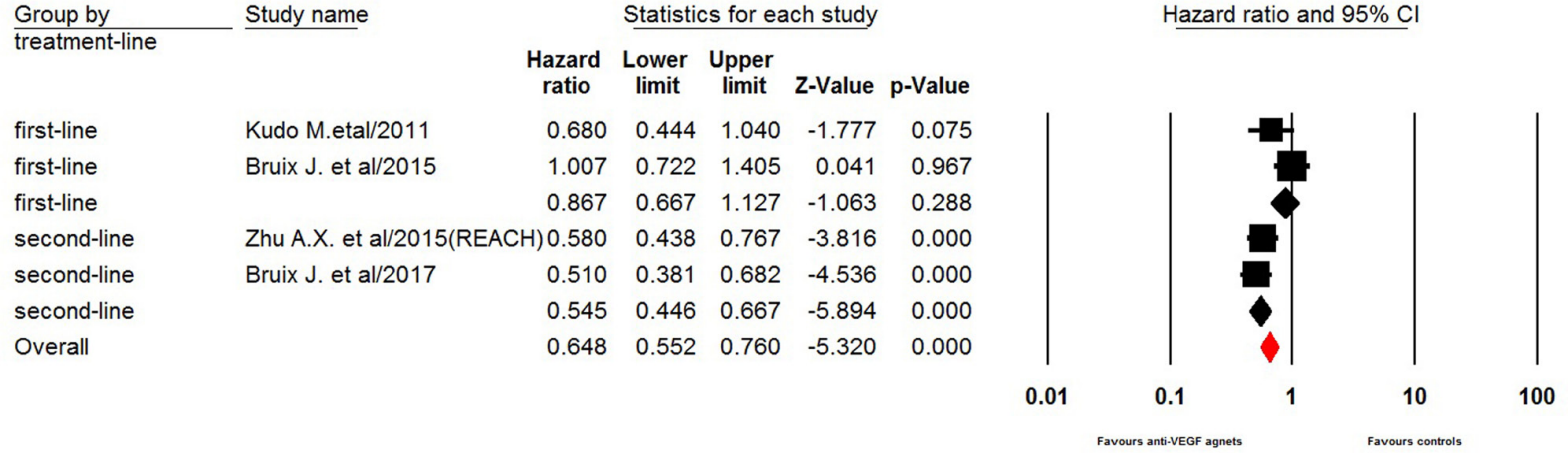
Fixed-effects model of hazard ratio (95% CI) of PFS associated with therapy with or without anti-VEGF agents

Four trials of the eight trials reported PFS data in the study patients. The pooled results demonstrated that the use of anti-VEGF agents significantly improved PFS in elderly HCC patients giving HR 0.65 (95% CI: 0.55–0.76, *p* < 0.001, Figure 2). Sub-group analysis according to treatment line showed that the use of anti-VEGF agents significantly improved PFS in elderly HCC patients who relapsed after a first-line sorafenib treatment (HR 0.55, 95% CI: 0.45–0.67, *p* < 0.001, Figure 2), while the use of anti-VEGF agents as first-line treatment did not significantly improved PFS in this patients population (HR 0.87, 95% CI: 0.67–1.13, *p* = 0.29). Begg's test and Egger's test revealed no evidence of obvious publication bias (*p* = 0.50 and *p* = 0.56, respectively).

### Overall survival

**Figure 3:**
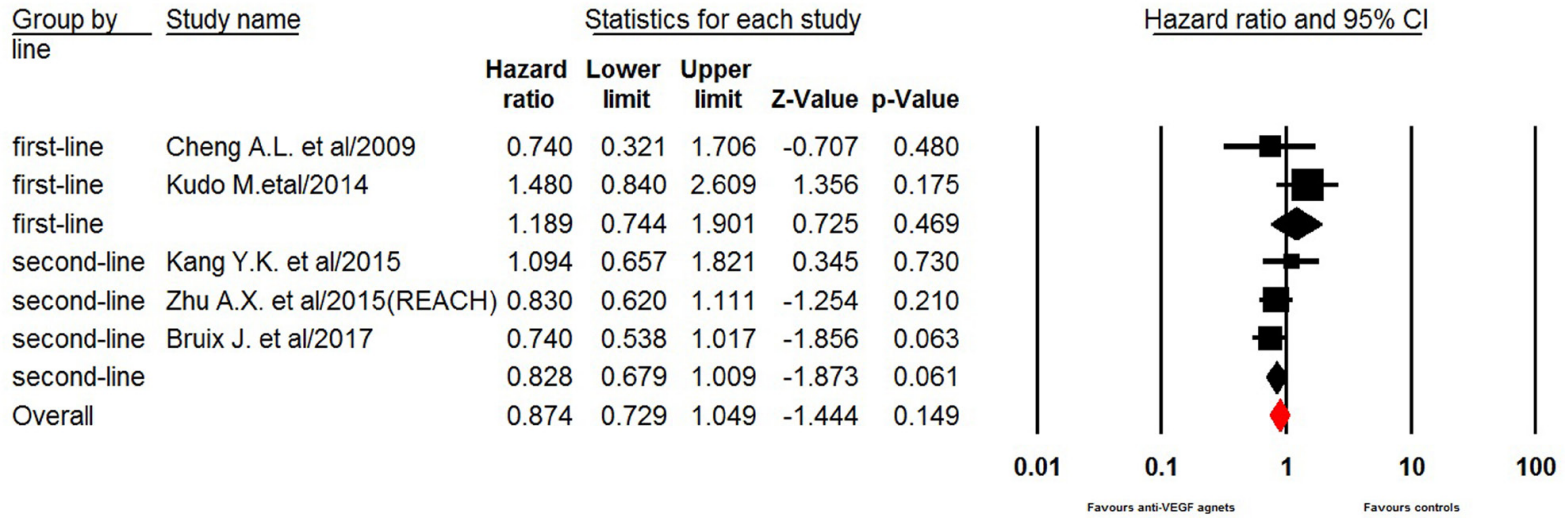
Fixed-effect model of hazard ratio (95%CI) of OS associated with therapy with or without anti-VEGF agents

Five of the seven trials reported OS data of elderly patients. Our pooled results demonstrated that the use of anti-VEGF agents did not significantly improved OS in elderly HCC patients giving HR of 0.87 (95% CI: 0.73–1.05, *p* = 0.15 Figure 3). However, sub-group analysis showed that the anti-VEGF agents rechallenge marginally improved OS in elderly (aged ≥ 65 years) HCC patients who previously treated with sorafenib (HR 0.83, 95% CI: 0.68–1.01, *p* = 0.061), while no survival benefit was obtained in elderly HCC received first-line anti-VEGF treatment (HR 1.19, 95% CI: 0.74–1.90, *p* = 0.47). Begg's test and Egger's test revealed no evidence of obvious publication bias (*p* = 0.33 and *p* = 0.38, respectively).

## DISCUSSION

In the past years, the mechanisms of hepato-carcinogenesis have been extensively investigated. Several tyrosine-kinase receptors, including vascular endothelial growth factor receptor (VEGFR), platelet-derived growth factor receptor (PDGFR), and the scatter growth factor receptor MET, have been implicated in the proliferation and invasion of HCC. HCC tumors are highly vascularized, and vascular endothelial growth factor (VEGF) augments HCC development and metastasis [39]. Vascular endothelial growth factor A (VEGF-A) promotes angiogenesis primarily through binding and activation of the receptor VEGFR-2 [40]. VEGF-A is frequently overexpressed in HCC, and is related with blood vessel density and tumor recurrence [40]. Makinen T. reported that VEGF-C/VEGFR-3 signaling pathway played a critical role in the growth and survival of lymphatic endothelial cells [41]. These findings suggest that inhibition of VEGF signaling pathway might be an effective strategy for the treatment of HCC patients. Sorafenib, a multikinase inhibitor of VEGFR, PDGFR-β, Raf, and other kinases has been shown to be efficacious against HCC, and has been approved as first-line treatment of advanced HCC. Several anti-VEGF agents also represent a promising treatment strategy to improve outcome of advanced HCC patients. A previous meta-analysis conducted by Niu M. et al. [42] showed that the use of anti-VEGF therapies in HCC patients significantly improved survival in comparison with placebo. However, there is limited data specifically focusing on the efficacy of anti-VEGF agents in elderly patients with HCC. As a result, we perform the present study to investigate the overall efficacy of anti-VEGF agents in the treatment of elderly HCC patients.

Our systematic review is, as far as we known, the first systematic review to specially assess the efficacy of anti-VEGF agents in the treatment of elderly HCC patients. Our study includes a total of 1,309 elderly (aged ≥ 65 years) HCC patients from seven trials. Our results demonstrate that the use of anti-VEGF agents MTAs in patients aged ≥ 65 years significantly improves PFS (HR 0.65, 95% CI: 0.55–0.76, *p* < 0.001) but not for OS (HR 0.87, 95% CI: 0.73–1.05, *p* = 0.15). Sub-group analysis according to treatment line shows that the use of anti-VEGF agents as second-line treatment significantly improves PFS (HR 0.55, 95% CI: 0.45–0.67, *p* < 0.001) and marginally improves OS (HR 0.83, 95% CI: 0.68–1.01, *p* = 0.061). Additionally, no survival benefits is observed in elderly HCC received first-line anti-VEGF treatments in terms of PFS (HR 0.87, 95% CI: 0.67–1.13, *p* = 0.29) and OS (HR 1.19, 95% CI: 0.74–1.36, *p* = 0.47). The findings of this study suggest that elderly HCC patients who relapsed after a first-line sorafenib treatment obtain a survival benefits from rechallenge use of anti-VEGF agents. Further studies are recommended to search for predictors of good responders in these patients received anti-VEGF agents.

Several limitations exist in this analysis. First of all, this is a meta-analysis at study level. We could not obtain individual patient data from the publication, thus we could not incorporate patients variables into the analysis. Second, there is moderate heterogeneity among the included studies, because different anti-VEGF agents are included for analysis. Additionally, the patient population in the present study is significantly heterogeneous, which might be another source of heterogeneity. However, clinical heterogeneity might improve the generalizability of the observed results. Third, none of the included trials report the toxicities of anti-VEGF agents in elderly patients. Thus, we could not answer whether the use of anti-VEGF agents in this patient population would increase the toxicities in comparison with controls. Finally, publication bias is an important issue in the meta-analysis. In the present, we detect no publication bias using Begg and Egger tests for OS and PFS.

## CONCLUSIONS

The findings of this study show that elderly HCC patients who relapsed after the first-line sorafenib treatment obtains a survival benefits from anti-VEGF agents rechallenge. Further studies are recommended to search for predictors of good responders in these patients received anti-VEGF agents.

## SUPPLEMENTARY MATERIALS TABLES




