# Supplementary material for: Efficacy of anti-VEGF agents in the treatment of elderly hepatocellular carcinoma: a systematic review
Source: Oncotarget. 2017 Oct 3;8(54):93179–85. doi: 10.18632/oncotarget.21452 (PMC5696253; doi:10.18632/oncotarget.21452)
Supplement: Supplementary file 1 [file oncotarget-08-93179-s001.pdf]

Efficacy of anti-VEGF agents in the treatment of elderly hepatocellular carcinoma: a systematic review

SUPPLEMENTARY MATERIALS

Supplementary Table 1: Search strategy for the systematic review

Search strategy used the following limits alone, or in combination: 1) terms describing cancer (“cancer”, “carcinoma”, and “neoplasm”); 2) terms describing liver tumor (“liver tumor”, “hepatocellular”, “hepatic”); 3) angiogenesis inhibitors (ie, “bevacizumab”, “sorafenib”, “vandetanib”, “pazopanib”, “aflibercept”, “cediranib”, “thalidomide”, “lenalidomide”, “sunitinib”, “axitinib”, “regorafenib”, “ramucirumab”); 5) therapy line (ie, “first-line”, “previously treated”, “refractory”, “second-line”); 6) clinical trials (ie, “prospective”, “clinical”, “human”, or “random”). In addition, we manually searched through abstracts submitted to the 2010 and 2016 American Society of Clinical Oncologists (ASCO) general meeting for applicable trials.

Supplementary Table 2: PRISMA 2009 Checklist. See Supplementary_Table_2
